# Identification of core competencies for primary care of allergy patients using a modified Delphi technique

**DOI:** 10.1186/1472-6920-11-12

**Published:** 2011-04-04

**Authors:** Joanna Wallengren

**Affiliations:** 1 Department of Clinical Sciences, Section of Dermatology, Lund University, Skane University Hospital, Lund, Sweden

## Abstract

**Background:**

The majority of allergy patients who seek medical advice are seen in primary care. In-service training of professionals in general practice is needed in order to increase knowledge among primary care clinicians about allergy. Therefore it is important to establish a consensus about what primary care professionals should be able to do, and what the public can expect. We sought to identify core competencies for good practice amongst primary care providers with respect to diagnosis and therapy of allergic diseases and to outline learning objectives for a postgraduate training programme in this field.

**Methods:**

The study involved three rounds, involving a total of 43 expert panellists. In the first round, a panel was asked to indicate competencies (knowledge, diagnostics, therapy and communication) necessary for primary care providers. The second and third rounds were answered by primary care physicians (26) and nurses (10). A Likert scale 1-4 was applied in the second round and two choices ("agree"/"disagree") in the third round, with a criterion of 75% being adopted.

**Results:**

The second round included 80 competencies and the third 50. The third round selected a consensus of 46 competencies defining nine learning outcomes for in-service medical training.

**Conclusions:**

The competencies in the field of allergy recommended in this study may serve as a reference of what can be expected from primary care providers.

## Background

Asthma, urticaria, atopic dermatitis, and allergic rhinitis are common diseases that impact the patient's quality of life and need for training and support. The number of allergy specialists varies in different parts of the world, and the majority of patients who seek medical advice are seen in primary care. Continuity of care is often easier to provide in general practice. Primary care has also been shown to be more cost-effective compared to hospital care [1]. There is, however, a great diversity in knowledge and tools for the management of this patient group in different settings and there is need for in-service training of professionals in order to provide guideline-driven and evidence-based health care. Recently, a few guidelines focused on management of allergy patients in primary care have been produced. So far, there are only guidelines for patients with respiratory allergy [2,3]. These guidelines are based on systemic reviews and expert opinions. The goal is that well trained primary care teams will be able to follow the guidelines. At many primary care units, allergy patients are treated by physicians and nurses with special expertise in asthma/COPD. These nurses have in-depth training in asthma and respiratory allergy. However, there may be gaps concerning manifestations of allergic diseases in other organs like skin or gastrointestinal tract. Therefore it is important to establish a consensus of what primary care professionals are able to do for all categories of patients with allergic diseases, and what the public can expect. As dermatologist, the author of the present study wanted to focus on a broad spectrum of allergic diseases, not only on the respiratory manifestations.

Competencies in primary care necessary for the management of allergy patients may be evaluated by different means. The most common approach is brainstorming or nominal group technique - structured face-to-face consensus meeting of specialists and general practitioners. A disadvantage of meetings is that some panel members can be inhibited by stronger individuals who dominate the group. Another approach for reaching consensus is a voting system at larger meetings [4]. The statements on which voting is carried out are prepared by the panel members but consensus is based on the vote. A pitfall here may be that the participants, although interested in the subject, do not always have extensive experience unless there are special criteria for voting.

A common approach for reaching consensus is the Delphi technique that has been employed for determining training needs and curriculum content of various medical undergraduate programmes as well as in-service professional training and health research [5-8]. In this procedure, the expert opinions are collected independently and anonymously using a postal system or e-mail [9]. Experts are invited to suggest competencies, without being aware of the identity of the other respondents. The suggestions are incorporated into a questionnaire that is sent to the panel participants who then complete the questionnaire anonymously in several rounds. As a part of the process, the responses are fed back in summarised form to the participants and the number of competencies is successively cut down by omitting statements that gained less then 25% agreement until consensus is reached. A recent report shows how a national Canadian paediatric trauma curriculum has been developed using the Delphi process. This curriculum was created from scratch by four experts who developed aims, objectives and skill sets through three rounds to reach consensus. The draft was later evaluated by 11 site coordinators and a final curriculum was established [10].

We sought to make recommendations towards core competencies in the field of allergy for primary care units. The reached consensus was then used to recommend learning objectives for postgraduate training. A modified Delphi technique was chosen for this project in order to obtain views from practitioners from across the country, giving each contributor equal weighting. As the primary care of allergy patients is often handled by a team, it was considered important to include nurses in the study.

## Methods

### Selection of panel members

The panel, recruited from the whole of Sweden, was invited to suggest what they considered important competencies in the primary care setting for investigation and treatment of allergy patients. Specialists in allergology (3), clinical immunology (3), dermatology (3), otolaryngology (3) and paediatric allergy (3) who are often engaged as lecturers in national training programmes in asthma and allergy and consensus meetings were contacted by e-mail. The reason for such selection was that specialists would be able to identify common practice gaps in their special fields of interest. However, only the professionals actually involved in primary care can see the reality for allergy patients among other patients treated at the unit. Therefore, the second and third rounds were performed by general practitioners and primary care nurses. The inclusion criteria for general practitioners with special interest in allergy (15) and primary care nurses with special expertise in asthma/COPD (5) were: professional experience exceeding five years and participation in educational programmes in the field of allergy. The final rating of the competencies was left to the doctors (39) and nurses (8) from primary care with special interest in allergy.

This study employs a modified Delphi methodology, keeping the core principle of elucidating most important issues by peeling off less important ones through several rounds. We were, however, not able to employ the same panel in all rounds of the investigation due to dropout as the respondents felt that participation was too time-consuming.

### Tools

The Delphi technique was carried out in three rounds. The aim of the first round was to list the competencies for good practice for primary care providers with respect to the diagnosis and therapy of allergic diseases. The first round took place in November and December 2008. The panellists were addressed with an open question, "What competencies in a primary care setting are desired for management of allergy patients?" and were asked to respond in their own words. The invitation was sent to 15 general practitioners, 5 asthma/COPD nurses and 15 secondary care physicians.

**Figure 1:**
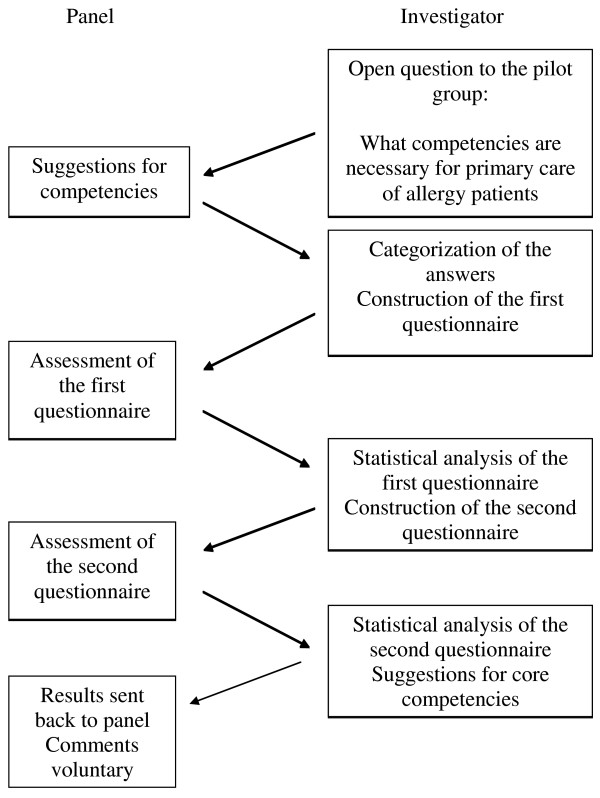
**Schematic description of the Delphi process in this study**.

All suggestions were categorised into four domains: knowledge, diagnostics, therapy and communication. Only repeated statements were omitted, and the remaining statements were incorporated into a questionnaire of 80 items. Statements that contained multiple concepts were split into different items. There was also the opportunity to submit other comments or suggestions and the respondents were encouraged to add competences not listed. This questionnaire was sent to the general practitioners and nurses in the second round in December 2008 and January 2009, with one reminder sent. The questionnaires were returned mainly by mail and were anonymous, although profession, number of years in the profession, number of hours spent with allergy patients a week and the gender of the respondent were stated. This questionnaire consisted of statements to be evaluated using a Likert scale 1-4: 1- not necessary, 2- desirable, 3- important, 4-necessary. Only statements scoring 3.25 points or more were incorporated into the next questionnaire, which was sent in the third round between January and February 2009. Once again there was an opportunity to submit other comments or suggestions and the respondents were encouraged to suggest and add competencies. This questionnaire was addressed to the same panel and to a further seven general practitioners, but no reminder was sent. In the third round, the questionnaire was quantitative - the listed statements were to be answered by: "agree"/"disagree". Responses of "do not know" were regarded as disagreement. An overview of the complete process in this study is shown in Figure 1.

### Data analysis

In the first questionnaire a mean score was calculated for all competencies listed. A minimum of 3.25 on the Likert scale was mandatory in order to qualify for inclusion in the second questionnaire in the third round. The cut-off point of 3.25 was an arbitrary decision as it corresponds to 75% of answers scoring 2 to 4 points (desirable - necessary). In the final round an agreement of 75% of the panel answers was necessary for inclusion of a competency in the final list, which was then regarded as consensus.

### Formulating the objectives of a training programme

A synthesis of core competencies into domains was used to specify the objectives of a training course to obtain the desired skills. In the present study, expected learning outcomes have been used to define the objectives for an educational programme [11]. The list of outcomes was supposed to serve as a framework for the organisation of learning resources and was to be used as an overview of the curriculum.

## Results

**Figure 2:**
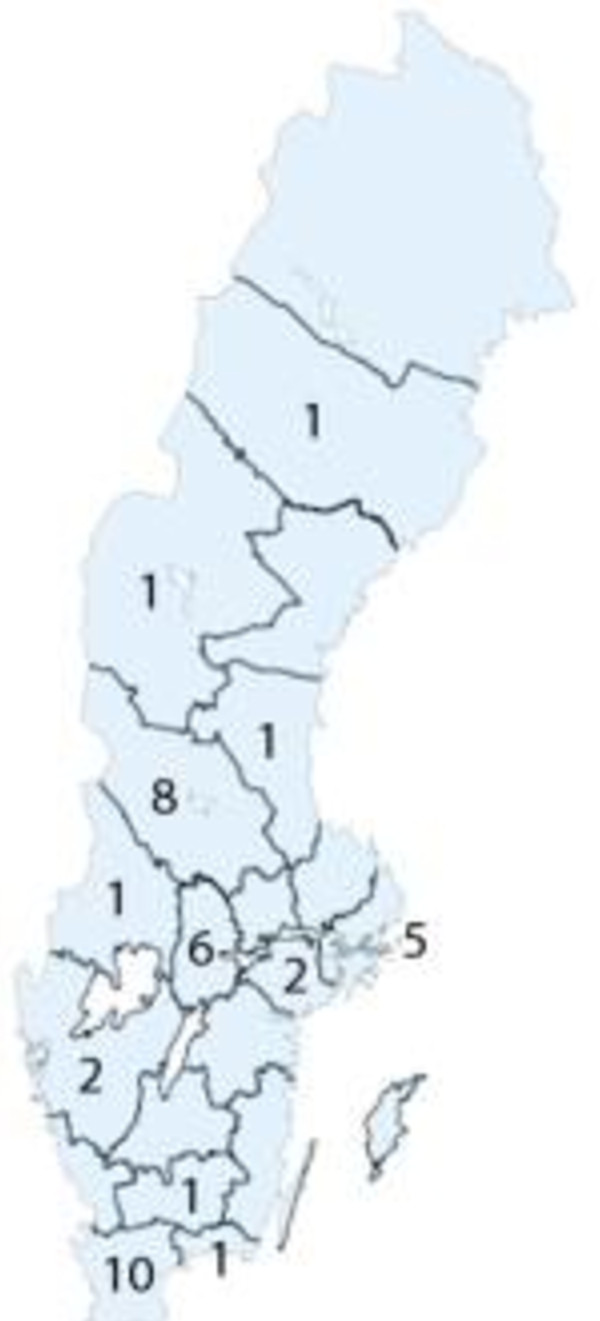
**The numbers of panel participants from the different regions of Sweden**.

A total of 35 individuals from the 12 biggest health regions in Sweden (out of 20) answered the questionnaires (Figure 2). The place of work of one participant could not be traced. Six primary care physicians and one nurse of total of fifteen invitees participated in the listing of the skills (Table 1). No striking differences were observed between the items generated by specialists versus generalists. Although all respondents were encouraged to suggest additional competencies in all rounds, no additional skills were generated by the second or third round.

**Table 1:** Classification of the Delphi participants

Category of expert	Invitees	Respondents	Respondents
	Round 1	Round 2	Round 3
Primary care physician	5	12	14
Primary care nurse	1	5	5
Secondary care physician	9		

### The first round of the investigation

Fifteen experts participated in listing the competencies: five general practitioners with special interest in allergy, one nurse with special expertise in asthma/COPD, three allergologists, one clinical immunologist, two dermatologists, two otolaryngologists and one paediatrician. One reminder e-mail was sent, and the response rate was 43%. Most participants came up with more than 10 suggestions for competences, although some generated just a few. Totally, the panel group came up with 80 different statements that were then categorised into four domains: knowledge (35), diagnostics (17), treatment (12) and communication (15). A few competencies occurred in more than one domain.

### The second round of the investigation

A questionnaire, comprising the data from the first round, was sent to 32 participants and was answered by twelve general practitioners (7 male, 5 female) and five nurses (all female) with special expertise in asthma/COPD. One reminder e-mail was sent and the response rate was 59%. The doctors worked approximately 1.3 hours a week (range 0.5-2 hours) with allergy patients and their mean professional experience was 17 years (range 6-28 y). The nurses worked approximately 7 hours a week (range 5-12 hours) with allergy patients and their mean professional experience was 15 years (range 7-20 y).

Thirty statements scored less than 3.25 and were excluded from the third round. These competencies are summarised in Table 2.

**Table 2:** Summary and priority of the items concerning knowledge or training in the second round that did not qualify for the third round

No	Statements with scores 2.3-2.5
1	Principles of elimination diets
2	Understanding of the difficulties in interpreting IgE and prick test reactions in children
3	Investigation of latex allergy
4	Occupational contact allergy
**No**	**Statements with scores 2.6-2.8**
5	The significance of specific IgE for the clinical symptoms of allergy
6	Occupational asthma and rhinitis
7	Occupational risks facing atopics
8	Different types of endogenous eczema
9	Mechanisms of pruritus
10	Indications for ASIT
11	Mechanisms of anosmia
**No**	**Statements with scores 2.9-3.1**
12	Understanding terms like sensitisation, atopy, type I and type IV allergy
13	Indication for and interpretation of specific IgE
14	Common food allergens in children and adults, hidden allergens
15	Indications for adrenaline autoinjector in children
16	Investigation of urticaria and pruritus
17	Trigger factors of chronic urticaria and eczema in children and adults
18	Signs of contact eczema and indications for patch test
19	Nurse with academic education in asthma at the unit

Fifty statements scored 3.25 or higher and were included in the third round of the study. These statements are listed in Table 3.

**Table 3:** The consensus for the core knowledge and skills for primary care of allergy patients

No	Knowledge	Agree(%)
1	Manifestations of allergy to pollen, mites and pets	100
2	Cross-reactions to birch and mugwort, oral allergic syndrome	79
3	Lactose intolerance	100
4	Trigger factors of acute urticaria/angioedema	95
5	Definition of anaphylaxis	95
6	Anaphylaxis from food, bee/wasp and drugs	95
7	Indications for prescription of adrenaline autoinjector	95
8	Intolerance vs allergy	100
9	What is included in different panels of allergens	100
10	Basic mechanisms of infectious asthma and COPD	100
11	Basic mechanisms of allergic and non-allergic rhinitis and organic disorders in the nose and sinus	95
12	Acute and chronic infectious disorders in the nose and sinus	95
13	Mechanisms of urticaria	90
14	Drug reactions: immediate and delayed	90
15	Basic mechanisms of common drugs for inflammatory disorders in airways and in skin	84
16	Different pharmacological treatment options (including the ones prescribed by specialists)	84
17	When to consider referral to a specialist	100
18	Not to advise avoidance of food due to occurrence of specific IgE in children without referral to a paediatric department	84
**No**	**Diagnostics**	**Agree(%)**
19	History, including seasonal allergies, cross-allergies; best with a questionnaire	100
20	Examination of eyes, nose and skin	100
21	Pulmonary auscultation	100
22	Basic investigation of obstructive pulmonary disease	100
23	Basic investigation of rhinitis	100
24	Basic investigation of type 1 and type 2 allergy	84
25	Indications of specific IgE	100
26	Reading of spirometry and PEF curve	100
27	Performing spirometry	79
28	Recognition of signs of contact allergy and referral to a dermatologist	100
**No**	**Therapeutics**	**Agree(%)**
29	Treatment of allergic rhinoconjunctivitis (antihistamine, nasal corticosteroid and eye drops)	100
30	Treatment of non-allergic disorders such as idiopathic or vasomotor rhinitis	100
31	Treatment and prevention of allergic acute asthma	100
32	Treatment of allergic acute asthma in children	90
33	Dealing with an acute severe allergic reaction	100
34	Treatment of anaphylaxis	100
35	Treatment of atopic eczema; moisturisers, topical steroids with adequate potency and avoidance of systemic steroids	100
36	Treatment of urticaria with non-sedating antihistamines	100
37	Treatment of itch	100
**No**	**Communication**	**Agree(%)**
38	Offering sufficient time to examine history	100
39	Showing interest, commitment and empathy	100
40	Writing careful referral for specific IgE	90
41	Explaining to the patient in a simple way what allergy is and its consequences	100
42	Giving basic advice about treatment of eczema and rhino-conjunctivitis	100
43	Instructing the patient how to use adrenaline autoinjector	79
44	Attending training courses as a team (doctor and nurse) to ensure patient receives consistent information	90
45	Cooperating with colleagues and other staff categories	100
46	Establishing regular cooperation with a specialist at the hospital	84

### The third round of the investigation

A questionnaire, generated by the data from the second round, was sent to the 32 original participants from primary care and to a further seven general practitioners with special interest in allergy. This questionnaire was answered by 14 doctors (8 males, 6 females) and five nurses (one male, four female). No reminder letter was sent and the response rate was 48%.

The doctors worked approximately 2.4 hours a week (range 1-6 hours) with allergy patients and their mean professional experience was 15 years (range 5-23 y). The nurses worked approximately 6.2 hours a week (range 2-12 hours) with allergy patients and their mean professional experience was 16 years (range 8-24 y). Only three experts participated in both questionnaires.

In this third round, only one statement (trigger factors of angioedema) was regarded as unnecessary by 25% of the respondents. All other competencies reached consensus. These competencies within the four domains are shown in Table 3.

### Specification of learning outcomes

A total of nine expected learning outcomes were extracted from the list of desired competencies (Table 4). The list indicates a desire for a holistic approach; not only examination and investigation of the patient but also understanding and communication of environmental risk factors as well as involvement and cooperation of different professionals in the care of the patient.

**Table 4:** Desired learning outcomes for in-service medical training in allergy for primary care settings

No	After the course the participants will be able to
1	Explain the mechanisms and manifestations of atopy, allergy, intolerance and autoimmunity and interpret the laboratory data
2	Examine the patient and investigate, treat and control asthma
3	Investigate and treat allergic and non-allergic reactions to food and drugs with special reference to principles for provocation
4	Diagnose and treat anaphylaxis
5	Investigate and treat allergic and non-allergic rhinitis and rhino-conjunctivitis
6	Investigate and treat different forms of urticaria and eczema with special reference to allergic and non-allergic mechanisms
7	Communicate with the patients about the risk factors, prevention strategies and treatment options with respect to the status
8	Identify criteria for referral to specialists, with special reference to paediatricians
9	Organise a team for continuing promotion of innovations in the care of allergy patients

110318

## Discussion

The Delphi technique to reach consensus among experts, using iteration of statements through several rounds, promotes reflection to arrive at the best possible solution. In the present study, only a few respondents participated in more than one round.

No secondary care physicians were asked to participate in the second and third rounds of this study as they were assumed to have insufficient knowledge about the facilities of primary care units. According to a questionnaire in 2000, 42% of the primary care units in the county of Stockholm employed general practitioners with special interest in asthma, and 58% employed asthma and COPD nurses (Kihlström A, personal communication). Naturally, the slight variation in the populations of the panel members in all three rounds is a limitation of this study and disqualifies it from being a pure Delphi study. It also highlights the main difficulty of this technique, namely the response rate. In the present study, the response rates in the second and third rounds were 59% and 48% respectively. Although some respondents were very enthusiastic about the project, especially those who accepted the invitation to the first round, some admitted that they lacked time to participate in projects like this. Probably the response rate could be increased if reminder letters were sent to non-responders [11].

The anonymous answers in the second and third rounds may guarantee honest opinions, but this anonymous way of collecting answers made it impossible to know who had actually responded.

The strength is the large number of participants from across the country, representing different geographical regions. In spite of the relatively low response rate (48-59%) it is assumed that we have obtained a balanced view. The structure of primary care, the distance to the nearest hospital, regional guidelines and in-service professional training vary in different parts of Sweden. This consensus includes possible regional differences as the participants were recruited from most regions of Sweden.

Normally in a Delphi study, the rounds should continue until all participants agree [9,12]. In the third round some competencies did not achieve full agreement. However, according to the Oxford Dictionary 1984, consensus is defined as "an agreement in opinion; a majority view" and 75% can thus be accepted as majority.

According to this study, most patients with moderate symptoms of asthma, food allergy, rhinitis, urticaria and atopic eczema can be treated in primary care settings. A referral to a paediatrician was recommended for children with food allergy and occurrence of specific IgE.

The general practitioner with special interest in asthma and allergy is responsible for diagnosis, treatment and follow up. A very important task is maintaining continuity and education of the patients on avoidance of allergens. The doctor and the nurse normally share this task and it was suggested that both should participate in the same in-service professional training in order to ensure consistency of information given to patients. One of the requirements suggested in the first round that did not make it to the third was academic education of nurses. In this study, all participating nurses had at least ten weeks of university education in the field of asthma and COPD. It may be expected that such education may result in better information to patients. Successful training of patients gives better understanding, compliance and safety, and results in fewer visits and admission to hospital [1]. Local knowledge of the environment at primary care units may also contribute to identifying important risk factors. Allergy-prevention programmes have been shown to reduce the prevalence of atopic symptoms in infancy [13].

Asthma and allergy nurses are normally responsible for spirometry and allergy testing. Prick tests are performed at some primary care units, and this is especially important where the secondary care institution is far away. A common view is, however, that frequent prick testing is required to maintain the high standard of the procedure, which is why many primary care providers do not perform it.

Spirometry is often performed in primary care units [14]. A "spirometry licensure", central education of key nurses at the primary care units, is now under discussion in Sweden in order to guarantee standard quality throughout the country. A primary care unit that treats children should have the competency to perform spirometry and interpret its results in this group according to the present survey.

In the future, increasing demands for quality assurance can require a licensure for primary care providers to treat allergic diseases. In the present study, expected learning outcomes have been used to define the objectives for an educational programme. Examination of primary care providers can also be used as a quality assurance. It seems that attendees of such a programme should have no difficulties in following guidelines on the management of asthma and allergic rhinitis [2,3]. The suggested competences and recommended learning objectives may set stage for a better implementation of guidelines; present and future, and for recognition of gaps in allergy management [15].

According to the present study, the training programme should be organised as a trans-professional course for physicians and nurses and include several practical elements. The importance of teamwork stressed by the panel suggests such a collaborative approach. Inter-professional education, defined as "occasions when members (or students) of two or more professions learn with, from and about one another to improve collaboration and the quality of care," has attracted increasing interest [16]. Such a coordinated educational programme for primary care providers in the field of allergy may also involve allergologists, clinical immunologists, dermatologists, otolaryngologists and paediatricians. It may constitute a good platform for better communication within the team of care providers, promoting better care of the patients.

## Conclusion

The most significant findings of the present study were importance of communication with the patients and special caution regarding children with allergy. Consequently, referral to a paediatrician was recommended for children with food allergy and occurrence of specific IgE. In addition to medical guidelines, training programmes should deal with communication skills. A trans-professional training programme was suggested in order to give the whole team the same recommendations, which would promote consistent information to the patients. It is possible that that this last reflection would have been captured by other means than the Delphi method and that the participation of nurses has contributed to give it the importance it deserves. Core competencies for good practice amongst primary care providers may constitute a reference for primary care of allergy patients and can be used for accreditation of primary care units.

## Competing interests

The author declares that they have no competing interests.

## Authors' contributions

JW conceived of, designed the study, analysed the data and drafted the manuscript.

## Pre-publication history

The pre-publication history for this paper can be accessed here:

http://www.biomedcentral.com/1472-6920/11/12/prepub
